# Chronic Polyaromatic Hydrocarbon (PAH) Contamination Is a Marginal Driver for Community Diversity and Prokaryotic Predicted Functioning in Coastal Sediments

**DOI:** 10.3389/fmicb.2016.01303

**Published:** 2016-08-19

**Authors:** Mathilde Jeanbille, Jérôme Gury, Robert Duran, Jacek Tronczynski, Jean-François Ghiglione, Hélène Agogué, Olfa Ben Saïd, Najwa Taïb, Didier Debroas, Cédric Garnier, Jean-Christophe Auguet

**Affiliations:** ^1^Equipe Environnement et Microbiologie, Institut Pluridisciplinaire de Recherche sur l’Environnement et les Matériaux, UMR 5254 CNRS - Université de Pau et des Pays de L’AdourPau, France; ^2^Laboratoire Biogéochimie des Contaminants Organiques, Unité Biogéochimie et Ecotoxicologie, Département Ressources Biologiques et Environnement, Ifremer Centre AtlantiqueNantes, France; ^3^Laboratoire d’Océanographie Microbienne, Sorbonne Universités, CNRS, Université Pierre-et-Marie-Curie, UMR 7621, Observatoire OcéanologiqueBanyuls-sur-mer, France; ^4^Littoral, Environnement et Sociétés, UMR 7266 CNRS – Université de La RochelleLa Rochelle, France; ^5^Laboratoire de Bio-surveillance de l’Environnement, Faculté des Sciences de BizerteZarzouna, Tunisia; ^6^Laboratoire Microorganismes: Génome et Environnement, UMR 6023 CNRS – Université Blaise PascalAubière, France; ^7^Processus de Transferts et d’Echanges dans l’Environnement, EA 3819, Université de ToulonLa Garde, France; ^8^Marine Biodiversity, Exploitation and Conservation, UMR CNRS 9190Montpellier, France

**Keywords:** microbial communities, PAH, chronic contamination, coastal sediment, functional diversity

## Abstract

Benthic microorganisms are key players in the recycling of organic matter and recalcitrant compounds such as polyaromatic hydrocarbons (PAHs) in coastal sediments. Despite their ecological importance, the response of microbial communities to chronic PAH pollution, one of the major threats to coastal ecosystems, has received very little attention. In one of the largest surveys performed so far on coastal sediments, the diversity and composition of microbial communities inhabiting both chronically contaminated and non-contaminated coastal sediments were investigated using high-throughput sequencing on the 18S and 16S rRNA genes. Prokaryotic alpha-diversity showed significant association with salinity, temperature, and organic carbon content. The effect of particle size distribution was strong on eukaryotic diversity. Similarly to alpha-diversity, beta-diversity patterns were strongly influenced by the environmental filter, while PAHs had no influence on the prokaryotic community structure and a weak impact on the eukaryotic community structure at the continental scale. However, at the regional scale, PAHs became the main driver shaping the structure of bacterial and eukaryotic communities. These patterns were not found for PICRUSt predicted prokaryotic functions, thus indicating some degree of functional redundancy. Eukaryotes presented a greater potential for their use as PAH contamination biomarkers, owing to their stronger response at both regional and continental scales.

## Introduction

Coastal ecosystems ecologically support numerous human activities (e.g., fishing, aquaculture, tourism, urban development, transport and refining of oil, industrial activities, etc.), which exert considerable anthropogenic pressure, potentially leading to the erosion of ecosystem health (Halpern et al., 2008; Borja et al., 2011). Hydrocarbon pollution constitutes the most significant threat, as it is estimated to represent worldwide between 1.3 and 8.8 million tons of discharge per year (National Research Council (US) Committee on Oil in the Sea, 2003). The ecological, economic and social repercussions of oil spills, such as the recent Deepwater Horizon oil spill in the Gulf of Mexico, are considerable. However, estimates of oil contamination show that oil spills are quantitatively less important than chronic pollution. For instance, they represent less than 30% of total input into the Mediterranean Sea (European Environment Agency, 2006). Additionally, coastal ecosystems receive significant and continuous inputs of pyrogenic hydrocarbons generated by combustion of fossil fuels (coal and oil) and other organic material such as wood (Page et al., 1999; Ravindra et al., 2008).

A huge diversity and density of *Bacteria* (Lozupone and Knight, 2007) and *Archaea* (Auguet et al., 2009), as well as a rich eukaryotic microfauna and meiofauna (Creer et al., 2010; Pernice et al., 2013), inhabit the marine sediment. Both prokaryotes and eukaryotes play pivotal roles in marine habitats. They support major ecosystem services like nutrient and organic matter cycling (Danovaro et al., 2008; Falkowski et al., 2008) and organic pollutant biodegradation, particularly hydrocarbons (Leahy and Colwell, 1990; Widdel and Rabus, 2001; Pérez-Pantoja et al., 2010; Coulon et al., 2012). The response of sediment microbial communities to acute PAH pollution events is very consistent across studies and usually characterized by a decrease in alpha-diversity due to the proliferation of hydrocarbonoclastic organisms (Head et al., 2006; Nogales et al., 2011; Kimes et al., 2014; King et al., 2015). However, the response to chronic pollution is largely unknown. It has recently been shown that pollution history influenced the response of coastal bacterial and nanoeukaryote communities to pollution (Sauret et al., 2012). Chronic pollution seems to lead to higher bacterial diversity, as a result of induced stability caused by long-term exposure (Nogales et al., 2007; Païssé et al., 2008; Zhang et al., 2008). However, several recent studies have shown a reduction of bacterial alpha-diversity in relation to elevated PAH concentrations (Rosano-Hernández et al., 2012; Sun et al., 2012, 2013; Korlević et al., 2015; Quero et al., 2015). *Deltaproteobacteria* and *Gammaproteobacteria*, which include several hydrocarbon degraders, are found to be dominant and recurrent in polluted and non-polluted sediments from coastal areas (Païssé et al., 2008; Zhang et al., 2008; Said et al., 2010; Sun et al., 2013; Quero et al., 2015). Compared to *Bacteria*, the response of *Archaea* to hydrocarbon contamination has received little attention (Röling et al., 2004; Dias et al., 2011; Stauffert et al., 2014; Sanni et al., 2015). Similarly, few studies have focused on the response of eukaryotic communities to sediment contamination by PAHs. Both fungi and algae play an important role in hydrocarbon degradation (Head et al., 2006; Prince, 2010; Coulon et al., 2012). For instance, fungi dominated the eukaryotic community in response to the Deepwater Horizon plume (Bik et al., 2012), indicating that benthic eukaryotic diversity and richness may be sensitive to hydrocarbon pollution (Johnston and Roberts, 2009). However, Chariton et al. (2010) showed that chronic pollution by hydrocarbons affected beta-diversity rather than the richness of both microfauna and meiofauna in estuarine sediments.

Understanding the behavior of highly diverse sediment communities in a complex habitat, where ecological, historical and anthropogenic processes operate at multiple spatial scales, is a complex task. To address this problem, we adopted a macro-ecological point of view and used a holistic approach in order to investigate the continental and regional patterns of communities from the three domains of life, in relation to hydrocarbon contaminations in coastal sediments from the North East Atlantic and Mediterranean regions. Based on *in silico* functional diversity inference, we additionally examined the relationship between prokaryotic functions and sediment contamination by PAHs.

## Materials and Methods

### Sediment Collection and Environmental Dataset

**FIGURE 1:**
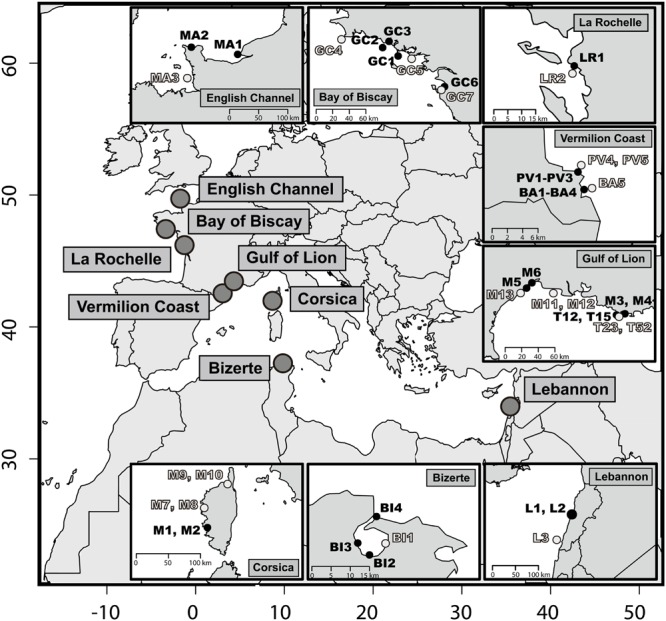
**Map of the 46 sediments sampling stations.** At each location *a priori* non-contaminated sites are in light gray and *a priori* contaminated sites in black. The *x*-axis and *y*-axis stand for decimal longitude and latitude, respectively.

Sediments were sampled at 46 sites across different geographical areas in order to be representative of the heterogeneity of the environment along the Atlantic and the Mediterranean coasts (**Figure 1**). Within each area, samples were collected from *a priori* PAH-contaminated and *a priori* uncontaminated sites according to site observations and the vicinities of potential hydrocarbon input. Among the polluted sampling sites, most samples were collected near to urbanized areas chronically impacted by shipping and industrial activities: Le Havre (samples MA1 and MA2), Lorient (GC1 and GC2), La Rochelle (LR1), Port-Vendres (PV1–PV3), Thau lagoon (M5 and M6), Toulon (M3, M4, T12, and T15), Beirut (L1 and L2), Bizerte (BI1), and Ajaccio (M1 and M2) (**Figure 1**). Certain *a priori* contaminated samples were also subjected to chronic pollution due to recreational shipping activities (BA1–BA4, Banyuls-sur-mer and GC1, Quiberon). Finally, sample GC6 was collected after the Erika oil spill (July 2000), which occurred in December 1999, while sample GC7 was collected before the Erika oil spill in August 1999 at the same sampling location. Coastal sediment samples were all taken from the surface sediment layer (1–2 cm), using a common sampling box corer. Toulon Bay samples (T12, T15, T23, and T52) have been described in detail by Tessier et al. (2011). The samples were frozen at –80°C within 1 day after sampling. Sediment particle size distribution was determined using laser diffraction, according to Tronczynski (2005) and Tessier et al. (2011). Total organic carbon (TOC) analyses were performed according to Tessier et al. (2011) and Azoury et al. (2013). TOC percentages (%TOC) and particle size distribution (% >63 μm; PSD) were available together for 32 of the 46 samples (Supplementary Table 1). Polyaromatic hydrocarbon (PAH) quantifications were made using gas chromatography coupled to mass spectrometry (GCMS), as described by Azoury et al. (2013) and Stauffert et al. (2013). In order to take the PAH partitioning and consequently the PAH bioavailability, into account in the sediment matrices, PAH concentrations were normalized to 1% of TOC (Burgess et al., 2000; Wenning, 2005). PAH isomeric ratios (or diagnostic ratios), Phenanthrene/Anthracene (P/A) and Fluoranthene/Pyrene (F/P) were calculated in order to determine the bulk origin of the PAH contamination in sediment samples. Anthracene and fluoranthene are produced during pyrosynthesis, and are less thermodynamically stable. Furthermore, the isomeres (i.e., same molecular formula but different chemical structure) of anthracene and fluoranthene, phenanthrene and pyrene, respectively, are rather formed during the catagenesis of organic matter leading to petroleum formation. P/A ratio values below 10 indicate a pyrogenic origin and F/P ratio values above 1 indicate pyrogenic sources of PAHs (Budzinski et al., 1997).

### Molecular Methods and Sequence Processing

Total genomic DNA (gDNA) was extracted from 250 mg of sediment samples using the PowerSoil DNA isolation kit (MO-BIO Laboratories Inc.). PCR amplifications were performed using primers 27f and 519r targeting the V1–V3 region of the bacterial 16S rRNA gene (Weisburg et al., 1991), primers 344f and 915r targeting the V3–V4 region of the archaeal 16S rRNA gene (Raskin et al., 1994) and primers 1560f and 2035r targeting the VR4 region of the eukaryotic 18S rRNA gene (Hardy et al., 2010). Two and half microliters of gDNA extract was used in a PCR reaction containing 4 mM MgCl_2_, 0.8 mM of dNTPs, 0.15 mM of each primer, and 1U of AmpliTaq Gold 360 DNA Polymerase (ThermoFisher Scientific), in a final volume of 30 μl. PCR was performed with initial denaturation at 95°C for 10 min, followed by 30 cycles at 95°C for 30 s, 53°C for 45 s and 72°C for 105 s, with a final extension of 72°C for 7 min. Pyrosequencing was performed at MR DNA - Molecular Research (Shallowater, TX, USA), using a Roche 454 GS-FLX Titanium instrument (Roche, NJ, USA). Pyrosequencing resulted in 693687, 290816, and 110044 raw reads for bacterial, archaeal and eukaryotic datasets, respectively (Supplementary Table 2). Raw reads from the 3 datasets were independently quality-based, trimmed and aligned on the May 2013 Greengenes reference alignment for prokaryotes (McDonald et al., 2012) and SILVA 119 database for eukaryotes (Quast et al., 2013). Operationnal taxonomic units (OTUs) were clustered at a 96% (*Bacteria*), 97% (*Archaea*), and 90% (*Eukarya*) identity threshold following the Mothur 454 Standard Operating Procedure (Schloss et al., 2009) with a few modifications. Bacterial OTUs were clustered at 96% identity threshold in order to obtain an unbiased richness compared to the full-length 16S rRNA gene (Kim et al., 2011). As the mean length of 18S rRNA gene sequences was 153 bp after trimming, taxonomic affiliations could not be defined at deep taxonomic levels for *Eukarya*, and eukaryotic OTUs were thus clustered at a 90% identity threshold. Eukaryotic taxonomic affiliations were determined as described by Taib et al. (2013). Sequence clustering resulted in 23280 bacterial OTUs, 4633 archaeal OTUs, and 1571 eukaryotic OTUs. All statistical analyses were performed on a random subsample of 3238, 829, and 804 sequences for bacterial, archeal and eukaryotic datasets, respectively, corresponding to the smaller number of sequences per sample in the datasets, after trimming and quality processing. The eukaryotic dataset encompassed 42 samples, and both prokaryotic datasets 46 samples. The complete data set was deposited in the NCBI Sequence Read Archive (SRA) database under study Accession no SRP063723.

Using ARB software (Ludwig et al., 2004), representative sequences of each prokaryotic OTU were aligned on the Greengenes ARB database (May 2013) and eukaryotic representative sequences were aligned on the 119 non-redundant releases in of the SILVA ARB database. Resulting phylogenetic trees were exported and used to run UniFrac weighted matrix calculation (Lozupone et al., 2006). Richness was computed using the R package phyloseq (McMurdie and Holmes, 2013). The mean phylogenetic diversity (PD) of 1000 random subsamples of each sample and phylogenetic species variability (PSV) were calculated with R.

### Functional Predictions

Predictions of metagenomic functions for *Bacteria* and *Archaea* were performed using a bioinformatic tool that predicts gene family abundances based on 16S rRNA gene surveys, using a database of phylogenetically referenced genomes (PICRUSt, Phylogenetic Investigation of Communities by Reconstruction of Unobserved States, Langille et al., 2013). PICRUSt is based on the correlation between phylogeny and functions and uses the phylogenetic proximity between public genomes and 16S rRNA OTUs. The first stage of the PICRUSt pipeline is pre-computed and consists of building a table of predicted KEGG (Kyoto Encyclopedia of Genes and Genomes; Kanehisa and Goto, 2000) Orthology (KO) counts of every taxon in the 16S reference tree, which also contains taxa with genomic data. The second stage consists of (i) normalizing the 16S OTU table by the predicted 16S rRNA gene copy number per cell, which is pre-computed in PICRUSt according to reference archaeal and bacterial genomes; and (ii) predicting metagenomic data from a biom table, which included taxonomic data from the 2013 issue of the Greengenes database and OTU table, based on the previous pre-computed files. Nearest Taxon Index (NSTI) scores, which are computed with metagenome prediction, are an indicator of the accuracy of the predictions, with lower values indicating a better accuracy. NSTIvalues were 0.19 ± 0.04 for *Bacteria* and 0.22 ± 0.07 for *Archaea* (mean ± SD, *n* = 46). These ranges of values are in agreement with values found for environmental samples (e.g., soil and hypersaline mats; Langille et al., 2013), indicating highly diverse communities and a lack of reference genomes for *Archaea*. Metagenomic prediction resulted in KO counts for each sample. Inferred KO counts were then grouped into KEGG PATHWAY maps (KEGG pathways). KEGG pathways unrelated to prokaryotic functions were manually discarded. KEGG pathways present in the datasets related to hydrocarbon biodegradation (*n* = 14 for *Bacteria* and *n* = 12 for *Archaea*) and related to organic matter metabolism and biosynthesis (*n* = 36) were sorted by hand according to their KEGG identification.

Although several studies have demonstrated congruencies between 16S rRNA based and functional gene phylogenies (Langille et al., 2013; Martiny et al., 2013; Aßhauer et al., 2015), we are aware that prediction approaches cannot replace whole metagenome or metatranscriptome profiling. The functional predictions made in this work are therefore considered only as an indication of the functional potential held in each community and not the ground-truth.

### Statistical Analysis

**FIGURE 2:**
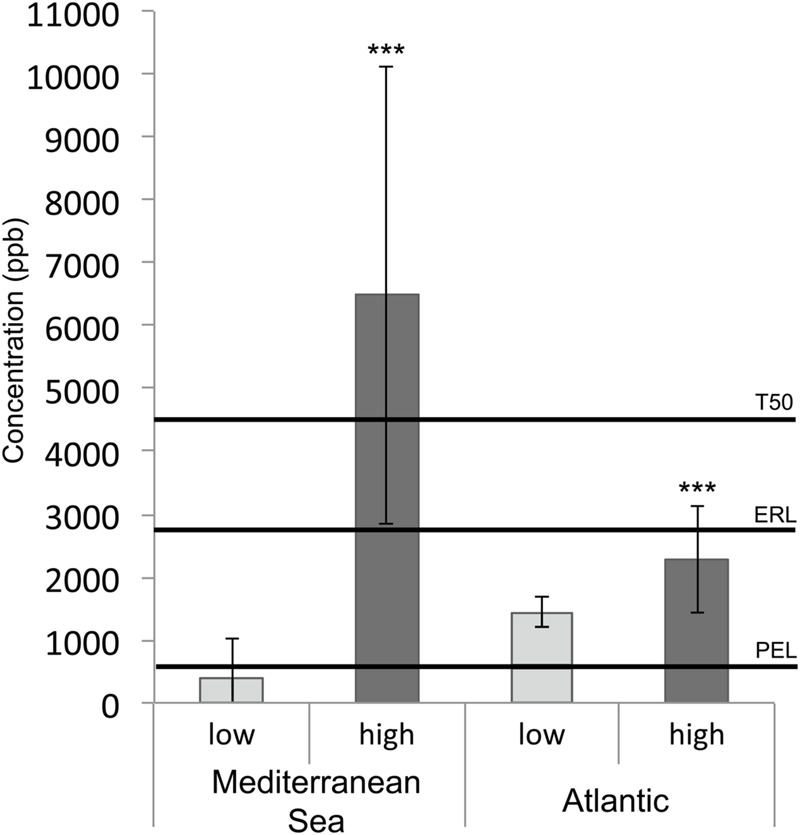
**Histogram presenting high and low PAH concentrations (μg/kg dw - sum of 9 individual compounds Σ PAH_9_: fluoranthene, pyrene, anthracene, ben[*a*]anthracene, benzo[*ghi*]perylene, chrysene, dibenz[*a,h*]anthracene, fluorene, indeno[1,2,3-*cd*]pyrene, phenanthrene) in sediment samples from the Mediterranean Sea and Atlantic coasts.** The asterisks indicate significant differences between highly contaminated and non-contaminated samples (*p* < 0.001). The plotted thresholds (solid line) correspond to Sediment Quality Guidelines: Probable Effect Level (PEL), Effect Range Low (ERL), and 50% toxicity concentration (T50).

The similarity of samples according to PAH profiles was assessed by calculating Euclidean distances based on 11 PAH concentrations and plotted as a dendrogram based on a hierarchical classification analysis (HCA, Supplmentary Figure 2). The Wilcoxon-Mann-Whitney *U* test or Student’s *t*-Test was used to compare mean total PAH concentrations between contaminated and non-contaminated samples from each geographic region.

Stepwise multiple regressions were performed on 32 samples, in order to investigate the relationships between alpha-diversity indices and environmental variables (concentrations of 11 individual PAHs, fluoranthene/pyrene and anthracene/phenanthrene ratios, temperature, salinity, and temperature of the water column, %TOC and PSD). Collinearities in the independent variables were tested before running the stepwise multiple regressions. Variables with collinearity up to 0.75 according to Spearman correlations (*p* < 0.05) were grouped together, and proxies of each group were used as explanatory variables (Supplementary Table 3). The PAH proxy concentration (i.e., dibenz[*a,h*]anthracene concentration) was log transformed, and the F/P ratio was squared transformed to improve the linearity and homoscedasticity of residuals, which were evaluated using the Shapiro-Wilk and Barlett tests, respectively. All statistical analyses were performed with R software.

Community similarity was represented by non-metric multidimensionnal scaling (NMDS) using the weighted UniFrac distance for data relating to 16S and 18S rRNA genes data and the Bray-Curtis dissimilarity for data relating to inferred KEGG pathways data (i.e., the whole prokaryotic dataset, hydrocarbons or organic matter related pathways). The phylogenetic structure was evaluated with the PSV index for each study (Helmus et al., 2007). PSV estimates PD as the variance of a trait evolving under a neutral model. The value is 1 when all species are phylogenetically unrelated (i.e., a star phylogeny) and approaches 0 as species become more related. To test statistically whether communities were composed of species that were related to each other to a greater or a lesser degree than expected, we compared the mean observed PSV with distributions of mean null values (1000 iterations) using two different randomization procedures. Null model 1 maintains species occurrence, whereas null model 2 maintains habitat species richness (Helmus et al., 2007). All these analyses were run with the R package picante (Kembel et al., 2010).

Mantel tests based on 9999 permutations were performed in order to test the correlations between Bray-Curtis matrices based on KEGG pathway composition, and UniFrac matrices. To assess the sources of variation (i.e., PAH concentrations, P/A and F/P ratios, latitude, PSD, %TOC, temperature and salinity) in both the phylogenetic and the functional distance matrix of the continental (i.e., Mediterranean and Atlantic data) and regional (i.e., Mediterranean or Atlantic data) datasets, we used a permutational multivariate analysis of variance (PerMANOVA) based on 1000 permutations (McArdle and Anderson, 2001) with the function adonis in the vegan package (Oksanen et al., 2013). Using the envfit function of the same package, significant environmental variables were fitted to the NMDS ordinations as vectors.

Biomarkers of environmental variation and hydrocarbon contamination were detected using the LEfSe algorithm (Segata et al., 2011). The first analysis step is a non-parametric Kruskal-Wallis (KW) sum-rank test allowing the detection of taxa with significant differential abundance. Biological consistency was subsequently investigated using a pairwise Wilcoxon test. Finally, Linear Discriminant Analysis (LDA) was used to estimate the effect size of each differentially abundant taxon. Alpha Values of 0.05 were used for KW and Wilcoxon tests and a threshold of 2.0 was used for logarithmic LDA scores. Significant differences between hydrocarbon-related KEGG pathways of contaminated and non-contaminated samples were tested using the *t*-test or the Wilcoxon test depending on the value distribution (i.e., normality of the data).

## Results

### Environmental Data

Salinity was higher at the Mediterranean sites (37.8 ± 0.8 g/l) than at the Atlantic sites (35.5 ± 2.2 g/l), and was highly correlated with latitude (rho = –0.87, *p*-value <0.001). Average temperature, PSD and %TOC were also significantly higher at the Mediterranean sites than at the Atlantic sites (Supplementary Figure 1). Within each geographic region, samples were collected in *a priori* contaminated areas like marinas and harbors, and in *a priori* uncontaminated areas (i.e., defined as uncontaminated prior to sampling). The concentrations of the 11 PAHs measured in this work were above detection limits for 44 of the 46 coastal sediment samples (Supplementary Table 1). As the Atlantic and Mediterranean sites were significantly different in terms of salinity, temperature, %TOC and PSD (Supplementary Figure 1), we analyzed the PAH gradient independently for each region. A HCA performed on the PAH concentrations showed that Mediterranean, and to a lesser extent Atlantic, sites were well separated according to our *a priori* contamination criterion (Supplementary Figure 2). PAH concentrations were significantly correlated (rho > 0.75, *p* < 0.05, Supplementary Table 3), allowing the calculation of the total PAH concentration for each sample and its comparison to the PAH sediment quality guidelines (SQGs) developed by Buchman (2008) (**Figure 2**): Probable Effect Level (PEL), Effect Range Low (ERL) and T50 (50% Toxicity Concentration). SQGs were available for 9 PAHs (fluoranthene, pyrene, anthracene, benz[*a*]anthracene, benzo[*ghi*]perylene, chrysene, dibenz[*a,h*]anthracene, fluorene, indeno[1,2,3-*cd*]pyrene, phenanthrene) among the concentrations of 11 PAHs measured for the whole sample set. The sum of these 9 PAHs (Σ PAH_9_) and the sum of the corresponding SQGs are shown in **Figure 2**. For both geographic regions, mean PAH concentrations were significantly higher in contaminated samples (*p* < 0.05), but the difference in PAH concentrations between non-contaminated and contaminated samples was much higher in the Mediterranean Sea (**Figure 2**), thus corroborating the results of the HCA. The mean of Σ PAH_9_ concentrations in highly contaminated samples from the Mediterranean Sea was significantly higher than the most conservative SQG with a value of 6483 μg.kg^-1^ (dry weight sediment; *p* < 0.05). In contrast, mean PAH concentrations in both non-contaminated and contaminated Atlantic samples were between PEL and ERL values, and consequently could not be distinguished regarding SQGs (**Figure 2**).

Fluoranthene to pyrene (F/P) and phenanthrene to anthracene (P/A) “source”-isomeric ratios are indicators of mixed-PAH origin (Budzinski et al., 1997; Neff et al., 2005). The values of the ratios indicates whether the PAH contamination originates more from direct input (petrogenic source) or from combustion processes (pyrogenic source). The ratio values suggested that the PAH contamination origin was predominantly pyrogenic in most of the samples, with P/A values lower than 10 and F/P values above 1 (Supplementary Figure 3; Supplementary Table 1). Therefore, our sediment samples were fairly representative of sites polluted by chronic PAH contamination, mainly due to from fossil fuel combustion, for most of the coastal areas studied. However, for 14 out of 44 samples, the indications given by the two ratios were contradictory, suggesting that both petrogenic and pyrogenic sources contribute to the contamination at these locations.

### Microbial Alpha-Diversity

**FIGURE 3:**
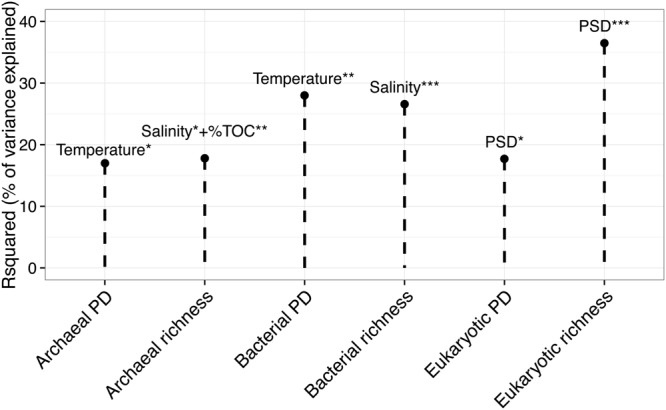
**Multiple linear regression model of richness and phylogenetic diversity (PD) of the three kingdoms (*x*-axis) against the set of explanatory variables.** The *y*-axis represents the determination coefficient (r-squared). Only significant regressions are shown. Asterisk corresponds to the level of significativity: ^∗^*p* < 0.05, ^∗∗^*p* < 0.01, ^∗∗∗^*p* < 0.001.

Prokaryotic 16S rRNA genes were successfully amplified for all the samples, while eukaryotic 18S rRNA genes were successfully amplified for 42 out of 46 samples. Using Richness (i.e., the number of OTUs per sample) and PD indices, we investigated the effect of PAHs on the alpha-diversity of the three domains of life. **Figure 3** shows the results of the stepwise multiple regression models of the two alpha-diversity indices against the explanatory variables. Temperature explained 28% and 17% of the variance of bacterial PD and archaeal PD, respectively. Salinity explained 27% of bacterial richness, and together with PSD, it explained 18% of archaeal richness. PSD significantly explained 37% of eukaryotic richness and 18% of eukaryotic PD. PAH concentrations and ratios were not found to significantly explain the variance of the alpha-diversity indices among the sites.

### Beta-Diversity Patterns

**FIGURE 4:**
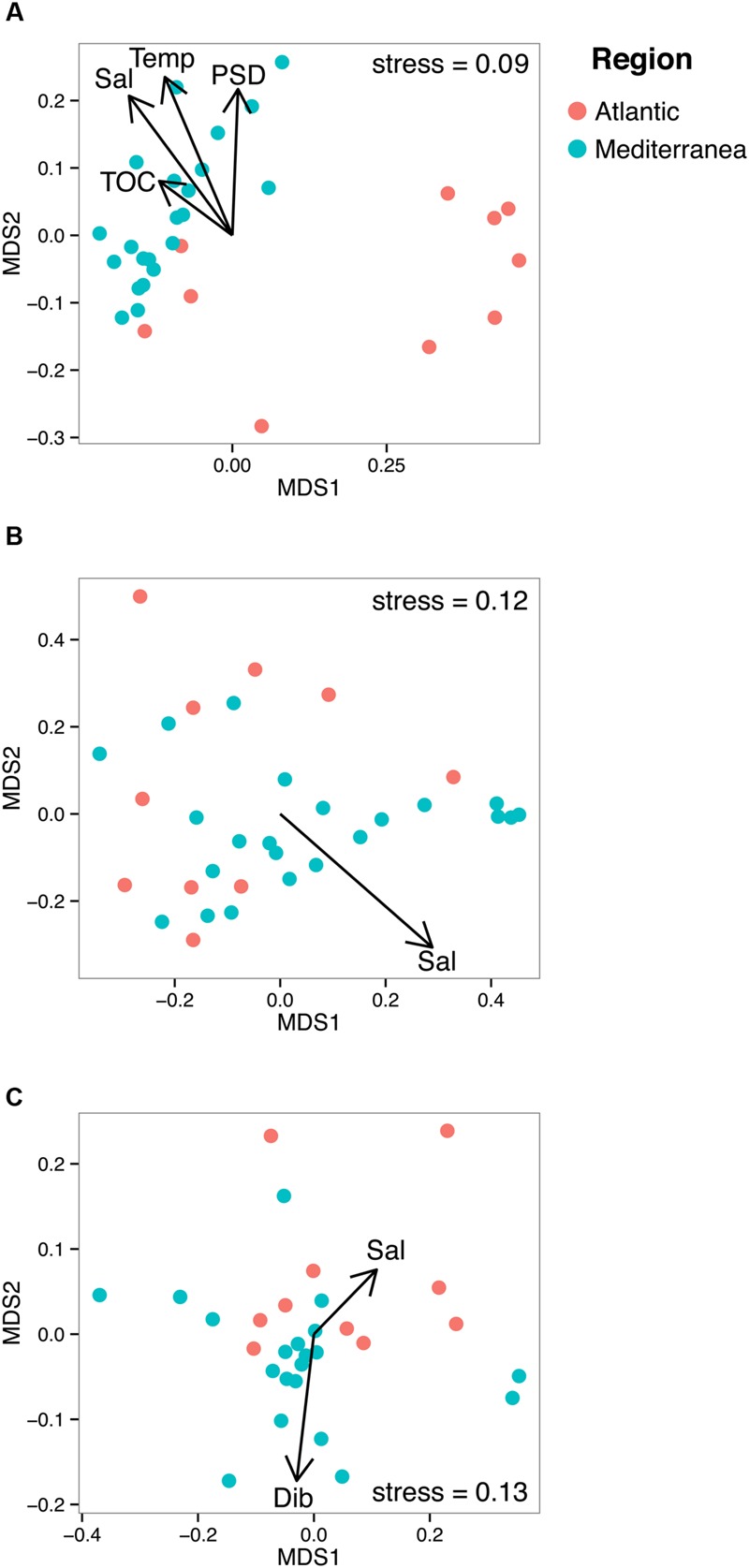
**Non-metric multidimensional scaling plots of bacterial **(A)**, archaeal **(B)**, and eukaryotic **(C)** UNIFRAC matrices.** The arrows represent the significant environmental variables fitted as vectors using the envfit function of the vegan package. Temp stands for temperature, Sal for salinity and Dib for Dibenz[*a,h*]anthracene.

In order to unveil a potential structuration effect of PAHs on microbial communities, these communities were sorted into an ordination plot according to their phylogenetic similarity (NMDS, **Figure 4**). For the three domains of life, the mean observed PSV values (0.66, 0.73, and 0.56 for *Bacteria, Archaea*, and *Eukarya*, respectively) were significantly lower (i.e., more phylogenetically clustered) than the null distribution for both model 1 (0.68, 0.74, 0.59, for *Bacteria, Archaea*, and *Eukarya*, respectively, *p* < 0.05) and model 2 (0.67, 0.76, and 0.57 for *Bacteria, Archaea*, and *Eukarya*, respectively, *p* < 0.05), indicating that sampling of phylotypes from the sequence pools was non-random. As non-random evolutionary processes were at work for both prokaryotes and eukaryotes, we tested their association with environmental parameters (i.e., salinity, temperature, %TOC particle size distribution, F/P and P/A ratios and dibenz[*a,h*]anthracene concentration). NMDS plots and PerMANOVA results revealed that the dissimilarities in the community phylogenetic structure of *Bacteria, Archaea*, and *Eukarya* were mainly driven by salinity (also a proxy of latitude), indicating the occurrence of different microbial communities at the regional scale (i.e., Atlantic *vs* Mediterranean, **Figure 4**; **Table 1**). The PerMANOVA analysis also showed that %TOC and particle size distribution (% <63 μm) significantly explained, respectively, 8.1% and 8.3% (*p* < 0.05) of bacterial communities phylogenetic variation, while no significant effect of these parameters was detected for the archaeal and eukaryotic domains. The habitat filter (i.e., selection of species by the local abiotic environment) therefore represented a strong structuring factor for the benthic communities of the three domains of life. The effect PAH concentrations was only detected for eukaryotic communities, for which 6.5% of the variation was explained by the PAH concentration proxy (i.e., dibenz[*a,h*]anthracene). When focusing on the Mediterranean or Atlantic regions, where environmental variability was lower, the structure of both bacterial and eukaryotic communities was significantly influenced by the P/A ratio (*R*2 = 9,3%, *p* < 0.05) and dibenz[*a,h*]anthracene (*R*2 = 11.8%, *p* < 0.05), respectively, (**Table 1**; Supplementary Figure 7). However, this tendency was observed only in Mediterranean samples, where the difference in PAH concentrations was maximal between polluted and unpolluted samples.

**Table 1:** PerMANOVA results for environmental parameters against UNIFRAC matrices.

	Bacteria		Archaea		Eukarya	
	*R*^2^	*P* value	*R*^2^	*P* value	*R*^2^	*P* value
***n* = 32**						
Salinity	18.9%	0.0001	7.4%	0.0399	6.5%	0.256
Temperature	13.8%	0.0011				
Particle size distribution (% <63 μm)	8.1%	0.03				
%Total Organic Carbon (TOC)	8.3%	0.0246				
Dibenzo[a,h]anthracene					6.1%	0.0394
***n* = 22 (Mediterranean Sea)**						
Particle size distribution (% <63 μm)	16.6%	0.0015				
Phenanthrene/Anthracene	9.3%	0.0471				
Dibenzo[a,h]anthracene					11.8%	0.0189
***n* = 10 (Atlantic)**						
Temperature	27.8%	0.0461				

*Non-significant parameters are not shown (*p* > 0.05). The percentages indicate the relative contribution of the parameters to matrix variation.*

**FIGURE 5:**
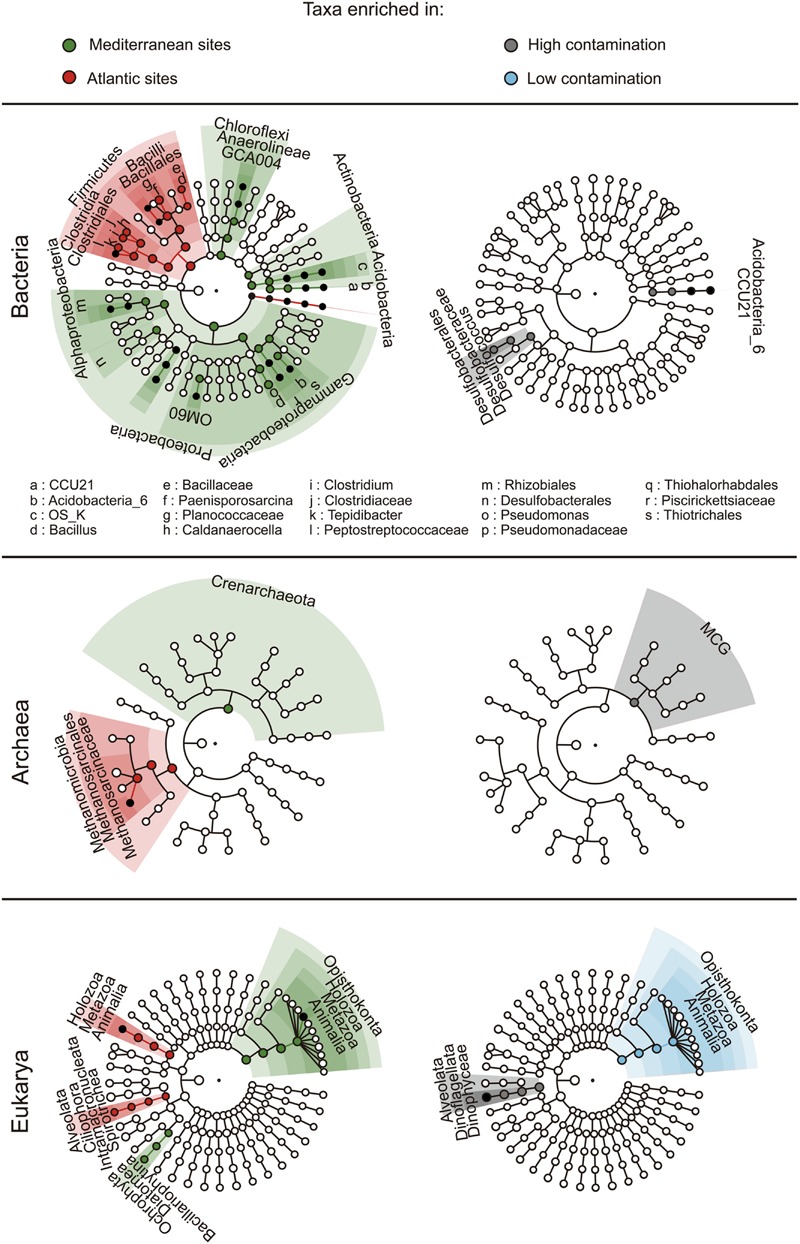
**LefSe analysis of the geographic origin (**left panel**) and the level of contamination (**right panel**).** The cladograms show taxa enriched (i.e., with significant differential abundance) within the environmental groups of samples (i.e., Mediterranean Sea and Atlantic; high and low contamination levels). The roots of the cladograms stand for the domain, and concentric circles represent the following taxonomic levels until the tips standing for genera. Biomarkers are shaded according to the groups of samples. The dot diameter is proportional to the abundance of the corresponding taxa.

The composition of the microbial communities was typical of marine benthic habitats with a domination of bacterial communities by *Deltaproteobacteria* (particularly of the *Desulfobacterales* order, Supplementary Figure 5) and *Gammaproteobacteria*, while archaeal communities were dominated by *Halobacteria* and *Thermoplasmata* and eukaryotic communities were dominated by *Annelida, Arthropoda*, and *Dinophyceae* (Supplementary Figures 4 and 5). We used the LEfSe algorithm in order to detect differentially abundant taxa between geographic regions and levels of contamination (i.e., the finding of environmental and hydrocarbon contamination biomarkers). LEfSe detected 78 taxa showing statistically significant and biologically consistent differences between geographic regions (**Figure 5**), with entire high taxonomic levels (i.e., aaa phylum level, and their respective taxonomic levels below) found as characteristic of Mediterranean (*Proteobacteria, Chloroflexi, Acidobacteria, Actinobacteria, Crenarchaeota*, and *Opisthokonta*) or Atlantic sediments (*Firmicutes and Holozoa*). In agreement with the PerMANOVA results showing an absence of influence of PAH contamination on the prokaryotic community structure at the continental scale, biomarkers of PAH contamination (i.e., with the presence and abundance statistically associated with the environmental condition) were rare with only 16 taxa detected. Among them, only *Archaea* of the *Miscellaneous Crenarchaeotic Group* (MCG) and eukaryotes from unclassified *Dinophyceae* to the superphylum *Alveolata* were found to be overrepresented in contaminated sediments (**Figure 5**).

### Prokaryotic Predicted Functional Diversity

The functional diversity of prokaryotic communities was reconstructed using PICRUSt. Overall, 60 and 55% of level 3 KEGG pathways (196 and 179 of 328 pathways) were present in the bacterial and archaeal datasets, respectively. Sources of variation related to environmental conditions in the Bray-Curtis dissimilarity matrices calculated on KEGG pathway abundances were explored by means of a PerMANOVA analysis (**Table 2**) and NMDS (Supplementary Figure 6). Similarly to the phylogenetic beta-diversity, the habitat filter represented a strong structuring factor for the reconstructed functional beta-diversity. Indeed, salinity, temperature and %TOC were the principal environmental variables shaping the structure of prokaryotic predicted functions whether we considered all functions, functions involved in organic matter degradation or functions related to hydrocarbon metabolism (**Table 2**). This is in agreement with the good correlation observed between the phylogenetic and functional distance matrices for *Bacteria* (*R*2 = 0.79, *p* = 0.0001) and *Archaea* (*R*2 = 0.66, *p* = 0.0001). No significant influences of the environmental parameters were detected when the Mediterranean and the Atlantic regions were considered independently. We then focused on the specific predicted KEGG pathway related to PAH degradation. Comparison of mean occurrences of the PAH degradation pathway between contaminated and non-contaminated samples in each geographic region (Mediterranean Sea and Atlantic) resulted in no significant difference for either *Archaea* or *Bacteria* (**Figure 4**). Overall, prokaryotic reconstructed metabolic potentialities were thus highly influenced by habitat, while the analysis did not detect any impact of PAH contamination.

**Table 2:** PerMANOVA results for environmental parameters against Bray-Curtis matrices of all reconstructed bacterial metabolic pathways, metabolic pathways (from the KEGG database) related to organic matter metabolism (OM, *n* = 113 for bacteria and *n* = 101 for archaea), and hydrocarbons (HC, *n* = 14 for Bacteria and *n* = 10 for Archaea).

	All pathways				HC related pathways				MO related pathways			
	Bacteria		Archaea		Bacteria		Archaea		Bacteria		Archaea	
	*R*2	*P* value	*R*2	*P* value	*R*2	*P* value	*R*2	*P* value	*R*2	*P* value	*R*2	*P* value
*n* = 32												
Salinity	51%	0.0001	11.20%	0.0342	50.5%	0.0001			50.8%	0.0001	11.70%	0.0289
Temperature	36%	0.0002	10.70%	0.0368	32.80%	0.0006			35.70%	0.0002	11.10%	0.0361
% TOC	17.5%	0.012			18.70%	0.0094			17.70%	0.0141		

*Non-significant parameters are not shown (*p* > 0.05). The percentages indicate the relative contribution of the parameters to matrix variation.*

## Discussion

In one of the largest surveys performed so far on coastal sediments, we adopted here a global approach, as opposed to experimental reductionist approaches, in which we characterized the microbial communities from the three domains of life, across a large PAH contamination gradient. This approach aimed to disentangle the impact of chronic PAH exposure from that of the habitat on microbial communities considering both the PD and the reconstructed metabolic potentialities.

### Effect of PAH Contaminations on Prokaryotic and Eukaryotic Alpha-Diversity

Microbial communities from coastal environments impacted by transitory input of hydrocarbons have been extensively studied during the past few years, especially following the Deepwater Horizon oil spill (Bik et al., 2012; Dubinsky et al., 2013; Kimes et al., 2013; Mahmoudi et al., 2013; Newton et al., 2013; Lamendella et al., 2014). An increase in the relative abundance of hydrocarbonoclastic bacteria following an oil spill is a very consistent result across studies (for reviews see Head et al., 2006; Kimes et al., 2014; King et al., 2015). The emergence of specialized taxa leads to the reduction of diversity within samples (Atlas et al., 2015). However, these profound changes in community composition are transitory (Ager et al., 2010; Mahmoudi et al., 2013) and a recovery of alpha-diversity has been reported in chronically or long-term hydrocarbon polluted areas (Gillan et al., 2005; Roesch et al., 2007). Generally, a decline in habitat health or a loss in ecosystem services results in an erosion of the richness or evenness of communities (Allison and Martiny, 2008). In contrast to what is observed in acute contamination events, we showed that the richness and PD of microbial communities in contrasted sedimentary coastal environments were not influenced by the chronic hydrocarbon pollution. This is typical of coastal microbial communities facing chronic pollution, and particularly bacterial communities (Hernandez-Raquet et al., 2006; Païssé et al., 2008; Zhang et al., 2008).

In contrast to prokaryote alpha-diversity, eukaryotic richness and PD were shown here to be highly related to particle size distribution, with higher richness and PD in coarser sediment. A greater richness in coarser sediments has been consistently observed in meiofauna and macrofauna assemblages, in both deep-sea and coastal sediments (Rodríìguez et al., 2003; McLachlan and Dorvlo, 2005; Netto et al., 2005; Hack et al., 2007; Leduc et al., 2012). Alternatively, no erosion of alpha-diversity indices could be attributed to PAH contamination, as previously shown by the comparisons between contaminated and non-contaminated sites (Chariton et al., 2010). However, as the sorption of PAHs on sediment matrices was not evaluated here, we cannot exclude the hypothesis that acute bioavailability of PAHs in finer sediments (Rockne et al., 2002; Ghosh et al., 2003) contributes, through an increase in toxicity, to the decline of eukaryotic richness in these finer sediments.

### Influence of PAH on Microbial Community Structure

Gillan et al. (2005) demonstrated that alpha-diversity alone is a poor indicator of ecosystem stress in chronically polluted systems, as the proliferation of new tolerant species can lead to the recovery of the diversity. Here, beta-diversity analyses revealed that the drivers of community structures where different depending on the scale of the dataset used. At the continental scale, and regardless of the domain of life, PAHs did not affect the microbial community composition compared to the selection pressure exerted by the natural environmental filter. In contrast, when considering the regional scale (i.e., the Mediterranean coast), the PAH contamination became the main driver of eukaryotic and bacterial communities variability, as the environmental filter was reduced and the range of PAH concentrations was larger. It is, however, important to note that most of the variance in community structure remained unexplained indicating that other regional environmental variables like nutrients or other contaminants (metals, antibiotics, etc.), may play an important role in shaping communities (Sun et al., 2012, 2013, Quero et al., 2015, Xie et al., 2016). Nonetheless, this result tends to agree with small-scales studies (i.e., covering few meters), which recorded changes in eukaryotic and bacterial community structures across similar PAH gradients (Païssé et al., 2008; Chariton et al., 2010; Païssé et al., 2010; Said et al., 2010; Sauret et al., 2015), although the changes were more subtle in the present work. Overall, the discrepancy between continental or regional datasets patterns gives an answer to a fundamental question raised by Nogales et al. (2011) by providing evidence that results from small scale studies may not be transposable to larger scales. Future risk assessment studies should therefore pay a particular attention to the scale of description when investigating the effect of contaminants on biological communities.

**FIGURE 6:**
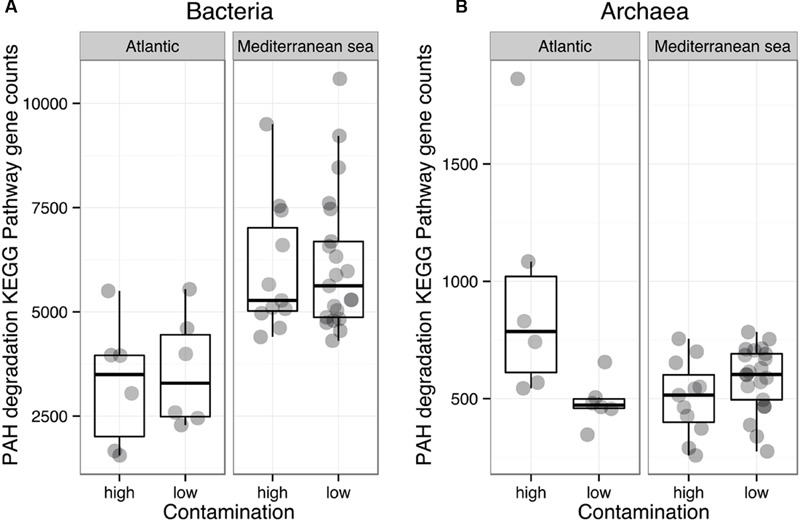
**Comparison of the mean abundances of bacterial **(A)** and archaeal **(B)** KEGG pathways related to the polyaromatic hydrocarbons (PAHs) degradation, within the Mediterranean and Atlantic geographic subsets**.

Although we acknowledge that functional assessment of microbial communities should involve the measurement of process rates (i.e., hydrocarbon degradation, prokaryotic production, enzymatic activities, etc.) (Rocca et al., 2015), we found that the subtle change in bacterial community composition was not reflected on predicted functions, indicating some degree of functional redundancy. Fundamental ecosystem services insured by sediment prokaryotic communities such as organic matter remineralization or hydrocarbon degradation did not seem to be altered by elevated PAH concentrations (**Figure 6**). Sun et al. (2012, 2013) found that PAHs were not as influential on bacterial communities as metal contaminants probably because of their lower toxicity due to their potential role as carbon source (Païssé et al., 2010), and potentially because of their high adsorption rate on sediment matrix, causing a decline of their bioavailability (Kraaij et al., 2002; Ghosh et al., 2003). Alternatively, a large diversity of known hydrocarbon degraders exists in the environment, particularly in *Bacteria* but also in *Archaea* and *Eukarya* (Al-Mailem et al., 2010; Prince, 2010; Tapilatu et al., 2010), and many of them have not yet been characterized (Nogales et al., 2011). *Deltaproteobacteria* and *Gammaproteobacteria*, which dominated unpolluted and polluted sites in this work are well known to be dominant clades in marine sediments and to encompass a large number of hydrocarbon degrader strains (Rojo, 2009; Kostka et al., 2011; Acosta-González et al., 2013). *Archaea* from the class *Halobacteria*, which dominated archaeal communities are also known hydrocarbon degraders (Al-Mailem et al., 2010). The diversity of prokaryotic degraders found here suggests that the benthic communities were naturally well adapted to face chronic hydrocarbon contamination, facilitating their resistance /resilience to this stress.

### Biomarkers of Chronic PAH Contaminations in Coastal Sediments

The identification of indicator species or biomarkers (i.e., a species or taxa lineage that define a trait or characteristic of the environment) is conceptually straightforward and has been widely used in ecological studies concerning both macro-organisms (Dufrêne and Legendre, 1997; De Cáceres et al., 2010; Pelletier et al., 2010) and microorganisms (Auguet et al., 2009; Chariton et al., 2010; Fillol et al., 2016). Here, community composition of the three domains of life was more stable across the PAH concentrations gradient than between geographic regions. Accordingly, fewer taxa indicative of PAH contamination were found, as compared to the taxa indicative of the geographic regions (**Figure 5**).

For *Bacteria*, the genus *Desulfococcus* and the taxonomic levels above (i.e., *Desulfobacteraceae* and *Desulfobacterales*) were identified as indicators of low PAH contaminations. The *Desulfobacterales* are particularly interesting, since the class to whom they belong (i.e., *Deltaproteobacteria*) is dominant in both polluted and pristine benthic marine ecosystems (Païssé et al., 2008; Leloup et al., 2009; Said et al., 2010; Korlević et al., 2015). This class also includes most of the sulfate-reducing genera, which play a crucial role in the anaerobic degradation of organic matter (Leloup et al., 2009) but also in hydrocarbon degradation (Suárez-Suárez et al., 2011; Stauffert et al., 2013). Members of the *Desulfobacterales* are dominant in the core microbiota of estuarine sediments (Sun et al., 2013). Therefore, a decline of the bacteria belonging to this order may negatively influence the rate of the processes they are involved in, and ultimately results in an important erosion of ecosystem health. However, genes involved in organic matter degradation pathways were not altered by high PAH levels potentially indicating a replacement by more tolerant SRB contributing to the functional redundancy of the system.

For *Archaea*, one taxon, the MCG was significantly linked to high levels of PAH contamination. MCG is a dominant and core archaeal taxa in sediment worldwide (Lloyd et al., 2013; Fillol et al., 2016). Recent findings from metagenomic data (Meng et al., 2014) and single cell genomics (Lloyd et al., 2013) suggested that members of this taxon might be involved in the degradation of aromatic compounds and detrital proteins, respectively. The former function may confer to members of the MCG lineage the capacity to use PAHs as source of carbon. This would be in agreement with their higher abundance in contaminated sediments.

Eukaryotic communities were the most impacted by PAHs both in terms of diversity and structure. Consequently, we were able to find more biomarkers related to PAH contamination for eukaryotic communities, than for *Bacteria* or *Archaea*. Taxa going from *Opisthokonta* to *Animalia* were significantly more abundant in non-polluted sediment, which is consistent with a potential toxicity of PAHs causing typically a decrease of macro-biota richness, characterized by the loss of sensitive species (Johnston and Roberts, 2009). In contrast, Eukaryotes from unclassified *Dinophyceae* taxa, and above until the superphylum *Alveolata*, were found to be overrepresented in contaminated sediments. Taxa belonging to the *Dinophyceae* class (dinoflagellates) were also detected as potential indicators of contaminated estuarine sediments (Chariton et al., 2010). We currently do not know whether the positive selection of *Dinophyceae* by PAHs is due to their capacity to metabolize hydrocarbons, or to their opportunistic ability to replace sensitive taxa. In any case, the increase of *Dinophyceae* in contaminated sediments may be of paramount importance since harmful algal blooms of benthic dinoflagellates cause major social, economical, environmental and health problems (European Environment Agency, 2006; Hoppenrath et al., 2013).

## Conclusion

Overall, our results showed that chronic PAH contamination was a marginal driver for coastal sediment community diversity, structure, and prokaryotic predicted functions compared to the habitat filter represented by salinity, latitude, particle size distribution and TOC. Nonetheless, the influence of the PAH contamination was greater especially for *Eukarya* at the scale of the Mediterranean coast. We provide evidence that results from studies focusing on the local scale may not be transposable to larger scales. Despite the weak response of prokaryotic and eukaryotic communities to PAH, we were able to identify potential biomarkers of chronic contamination. Owing to their higher sensitivity to PAHs, eukaryotes showed the greatest potential as biomarkers in Mediterranean and Atlantic sediments among the three domains of life, both at the regional and continental scales.

## Author Contributions

J-CA, JG, and RD conceived and designed the study. MJ ran the experiments. MJ, J-CA, JG, NT, and DD analyzed the resulting data. J-FG, JT, OS, HA, and CG provided samples, chemical and physical analysis. MJ and J-CA wrote the manuscript. J-FG, JT, HA, CG, and RD revised the manuscript.

## Conflict of Interest Statement

The authors declare that the research was conducted in the absence of any commercial or financial relationships that could be construed as a potential conflict of interest.
