# Supplementary material for: Chronic Polyaromatic Hydrocarbon (PAH) Contamination Is a Marginal Driver for Community Diversity and Prokaryotic Predicted Functioning in Coastal Sediments
Source: Front Microbiol. 2016 Aug 19;7:1303. doi: 10.3389/fmicb.2016.01303 (PMC4990537; doi:10.3389/fmicb.2016.01303)
Supplement: Supplementary file 1 [file Data_Sheet_1.DOCX]

Supplementary Material

Chronic PAHs contamination is a marginal driver for community diversity and prokaryotic predicted functioning in coastal sediments

Mathilde Jeanbille*, Jérôme Gury, Robert Duran, Jacek Tronczynski, Jean-François Ghiglione, Hélène Agogué, Olfa ben Saïd, Najwa Taïb, Didier Debroas, Cédric Garnier and Jean-Christophe Auguet

*** Correspondence:** Corresponding Author: mathilde.jeanbille@univ-pau.fr

# Supplementary table

**Supplementary table 1 :** Geographical description, environmental parameters, PAH concentrations (ppb) and ratios. ND = Not Determined.

|  |  |  |  |  |  |  |  |  |  |
| --- | --- | --- | --- | --- | --- | --- | --- | --- | --- |
|  | **Geographic areas** | **Longitudes** | **Latitudes** | **Month and year of sampling** | **Water column depth (m)** | **Water column salinity (g/l)** | **Water column temperature (°C)** | **Granulometry (%>63µm)** | **% TOC** |
| **BA1** | **Vermilion Coast** | 3.135056 | 42.490889 | 04-2012 | 6 | 37.8 | 16.4 | 43.3 | 6.1 |
| **BA2** |  | 3.135056 | 42.490889 | 07-2012 | 6 | 37.8 | 16.4 | 6.7 | 1.7 |
| **BA3** |  | 3.135056 | 42.490889 | 11-2012 | 6 | 37.8 | 16.4 | 24.3 | 4.5 |
| **BA4** |  | 3.135056 | 42.490889 | 02-2013 | 6 | 37.8 | 16.4 | 20.7 | 3.7 |
| **BA5** |  | 3.149167 | 42.491917 | 04-2012 | 24 | 37.8 | 16.4 | 5.8 | 1.5 |
| **PV1** |  | 3.120583 | 42.521639 | 04-2012 | 14 | 37.8 | 16.4 | 52.1 | 7.0 |
| **PV2** |  | 3.120583 | 42.521639 | 07-2012 | 14 | 37.8 | 16.4 | 57.1 | 6.0 |
| **PV3** |  | 3.120583 | 42.521639 | 02-2013 | 24 | 37.8 | 16.4 | 11.2 | 2.9 |
| **PV4** |  | 3.125278 | 42.530583 | 11-2012 | 14 | 37.8 | 16.4 | 59.3 | 6.3 |
| **PV5** |  | 3.125278 | 42.530583 | 04-2012 | 24 | 37.8 | 16.4 | 3.8 | 2.1 |
| **BI1** | **Bizerte** | 9.926008 | 37.186503 | 04-2012 | 2 | 29 | 23.7 | ND | 5.1 |
| **BI2** |  | 9.838863 | 37.141826 | 04-2012 | 2 | 28.8 | 23.2 | ND | 4.1 |
| **BI3** |  | 9.785814 | 37.187591 | 04-2012 | 2 | 27 | 23.1 | ND | 2.1 |
| **BI4** |  | 9.873962 | 37.266868 | 04-2012 | 2 | 32.2 | 23.4 | ND | 3.5 |
| **L1** | **Lebannon** | 33.916163 | 35.544857 | 07-2007 | ND | 39.3 | 22.5 | ND | ND |
| **L2** |  | 33.916163 | 35.544857 | 07-2007 | ND | 39.3 | 22.5 | ND | ND |
| **L3** |  | 33.263700 | 35.082110 | 07-2007 | 300 | 39.3 | 22.5 | 100.0 | 0.9 |
| **M3** | **Gulf of Lion** | 5.909040 | 43.083000 | 02-2004 | 8.5 | 37.9 | 17 | 61.8 | ND |
| **M4** |  | 5.909040 | 43.083000 | 07-2010 | 8 | 37.9 | 17 | 78.9 | 6.0 |
| **M5** |  | 3.380500 | 43.242900 | 05-2004 | 12 | 37.5 | 16.5 | 78.5 | 4.2 |
| **M6** |  | 3.571333 | 43.452800 | 04-2006 | 2.2 | 37.5 | 16.5 | 90.8 | 3.6 |
| **M11** |  | 4.402219 | 43.404000 | 08-2005 | 20 | 35.5 | 17.7 | 75.0 | 1.4 |
| **M12** |  | 4.402219 | 43.404000 | 08-2010 | 17 | 35.5 | 17.7 | 77.7 | 0.9 |
| **M13** |  | 3.571333 | 43.452800 | 05-2004 | 6 | 37.5 | 16.5 | 23.0 | 6.7 |
| **T12** |  | 5.916667 | 43.100000 | 06-2009 | 14 | 37.8 | 19.8 | 72.0 | 4.2 |
| **T15** |  | 5.900000 | 43.083333 | 10-2009 | 11 | 37.6 | 19.6 | 60.0 | 5.0 |
| **T23** |  | 5.944950 | 43.100867 | 10-2009 | 21 | 37.7 | 19.9 | 50.8 | 4.9 |
| **T52** |  | 5.966667 | 43.066667 | 10-2009 | 61 | 38 | 20.3 | 77.6 | 3.0 |
| **M1** | **Corsica** | 8.754998 | 41.920000 | 03-2004 | 65 | 38.1 | 18.2 | 67.8 | ND |
| **M2** |  | 8.754998 | 41.920000 | 07-2010 | 37 | 38.1 | 18.2 | 7.9 | 2.2 |
| **M7** |  | 8.670991 | 42.277000 | 03-2004 | 87 | 38.1 | 18.2 | 68.9 | ND |
| **M8** |  | 8.670991 | 42.277000 | 07-2010 | 91 | 38.1 | 18.2 | 75.5 | 4.1 |
| **M9** |  | 9.299997 | 42.731000 | 03-2004 | 99 | 38.1 | 13.5 | 39.4 | ND |
| **M10** |  | 9.299997 | 42.731000 | 07-2010 | 96 | 38.1 | 13.5 | 36.5 | 1.3 |
| **GC1** | **Bay of Biscay** | -3.074845 | 47.496700 | 06-1999 | 12.2 | 34.8 | 13.5 | 33.4 | 0.8 |
| **GC2** |  | -3.428251 | 47.666700 | 06-1999 | 27.6 | 34.8 | 13.5 | 46.9 | 3.1 |
| **GC3** |  | -3.365356 | 47.703700 | 06-1999 | 20.2 | 34.8 | 13.5 | 20.5 | 0.9 |
| **GC4** |  | -2.791339 | 47.454000 | 06-1999 | 74.4 | 34.4 | 13 | 55.6 | 1.0 |
| **GC5** |  | -2.791339 | 47.454000 | 06-1999 | 14.2 | 34.4 | 13 | 40.5 | 1.1 |
| **GC6** |  | -2.015060 | 47.010600 | 07-2000 | 0 | 33.7 | 13.5 | 51.2 | 2.6 |
| **GC7** |  | -2.015100 | 47.010600 | 08-1999 | 0 | 33.7 | 13.5 | 38.1 | 1.9 |
| **MA1** | **English channel** | -0.276001 | 49.505300 | 06-2009 | 16 | 34.8 | 11.7 | 8.9 | 0.9 |
| **MA2** |  | -1.638377 | 49.672100 | 06-2009 | 15 | 34.8 | 11.7 | 5.6 | 0.6 |
| **MA3** |  | -1.776359 | 48.949000 | 07-2001 | 12.3 | 35 | 12.3 | 26.7 | 2.7 |
| **LR1** | **La Rochelle** | -1.166972 | 46.145660 | 10-2013 | 2 | 33.6 | 19 | ND | ND |
| **LR2** |  | -1.162000 | 46.122000 | 10-2013 | ND | 33.6 | 19 | ND | ND |

**Supplementary table 1** (continued)

|  |  |  | **Bulk PAHs concentrations (ppb)** | | | | | | | | | | |
| --- | --- | --- | --- | --- | --- | --- | --- | --- | --- | --- | --- | --- | --- |
|  | **Phenanthrene/Anthracene** | **Fluoranthene/Pyrene** | **Fluoranthene** | **Pyrene** | **Anthracene** | **Benz[*a*]anthracene** | **Benzo[*a*]pyrene** | **Benzo[*ghi*]perylene** | **Chrysene** | **Dibenz[*a,h*]anthracene** | **Fluorene** | **Indeno[1,2,3-*cd*]Pyrene** | **Phenanthrene** |
| **BA1** | 2.76 | 1.33 | 231 | 173 | 59 | 208 | 285 | 107 | 106 | 33 | 39 | 236 | 162 |
| **BA2** | 5.18 | 1.38 | 96 | 70 | 11 | 28 | 29 | 24 | 22 | 4 | 6 | 29 | 57 |
| **BA3** | 2.91 | 0.70 | 56 | 79 | 20 | 85 | 91 | 36 | 36 | 11 | 13 | 74 | 58 |
| **BA4** | 3.14 | 0.86 | 471 | 545 | 144 | 496 | 421 | 165 | 169 | 44 | 79 | 307 | 453 |
| **BA5** | 4.68 | 1.28 | 14 | 11 | 3 | 9 | 17 | 11 | 7 | 2 | 1 | 18 | 12 |
| **PV1** | 2.06 | 1.19 | 869 | 728 | 161 | 1072 | 1318 | 443 | 471 | 151 | 42 | 976 | 332 |
| **PV2** | 2.47 | 1.32 | 119 | 91 | 24 | 106 | 116 | 57 | 40 | 19 | 9 | 122 | 59 |
| **PV3** | 4.58 | 1.45 | 99 | 69 | 1 | 70 | 74 | 33 | 29 | 10 | 15 | 69 | 5 |
| **PV4** | 2.76 | 1.26 | 793 | 630 | 106 | 700 | 954 | 362 | 251 | 96 | 41 | 703 | 294 |
| **PV5** | 3.36 | 1.23 | 20 | 16 | 2 | 17 | 12 | 12 | 10 | 4 | 1 | 28 | 7 |
| **BI1** | 9.44 | 0.58 | 8 | 13 | 3 | 2 | 3 | 8 | 2 | 5 | 4 | 4 | 24 |
| **BI2** | 4.80 | 0.74 | 66 | 90 | 25 | 31 | 42 | 78 | 22 | 19 | 56 | 74 | 118 |
| **BI3** | 3.28 | 1.24 | 110 | 89 | 25 | 60 | 51 | 64 | 52 | 31 | 30 | 162 | 81 |
| **BI4** | 8.98 | 0.64 | 2 | 4 | 1 | 1253 | 969 | 734 | 582 | 166 | 24 | 644 | 5 |
| **L1** | 8.39 | 0.46 | 20 | 42 | 6 | 22 | 13 | 30 | 43 | 7 | 39 | 25 | 47 |
| **L2** | 4.47 | 0.57 | 16 | 29 | 5 | 16 | 10 | 22 | 22 | 13 | 15 | 19 | 20 |
| **L3** | 7.78 | 1.07 | 6 | 6 | 1 | 3 | 3 | 4 | 4 | 1 | 1 | 5 | 6 |
| **M3** | 2.97 | 0.81 | 1198 | 1482 | 217 | 1065 | 1293 | 775 | 1304 | 234 | 32 | 849 | 645 |
| **M4** | 4.60 | 1.22 | 1540 | 1260 | 111 | 870 | 990 | 716 | 934 | 160 | 1 | 822 | 511 |
| **M5** | 3.96 | 1.12 | 1042 | 933 | 124 | 544 | 696 | 549 | 674 | 140 | 51 | 565 | 493 |
| **M6** | 3.97 | 1.34 | 1640 | 1220 | 83 | 672 | 694 | 545 | 666 | 144 | 18 | 726 | 331 |
| **M11** | 13.33 | 1.20 | 12 | 10 | 1 | 6 | 4 | 5 | 12 | 5 | 4 | 5 | 16 |
| **M12** | 14.30 | 1.32 | 15 | 11 | 1 | 5 | 4 | 9 | 9 | 5 | 1 | 6 | 14 |
| **M13** | 5.83 | 1.12 | 57 | 51 | 6 | 32 | 37 | 33 | 36 | 9 | 4 | 38 | 34 |
| **T12** | 5.57 | 1.10 | 4020 | 3660 | 460 | 1730 | 1850 | 1360 | 1960 | 150 | 220 | 1930 | 2560 |
| **T15** | 4.62 | 1.13 | 1170 | 1040 | 130 | 600 | 690 | 600 | 560 | <50 | 50 | 1340 | 600 |
| **T23** | ND | ND | <34 | <32 | <5 | <15 | <19 | <16 | <15 | <50 | <10 | <29 | <17 |
| **T52** | ND | ND | <34 | <32 | <5 | <15 | <19 | <16 | <15 | <50 | <10 | <29 | <17 |
| **M1** | 1.44 | 0.93 | 1300 | 1405 | 369 | 933 | 1305 | 1165 | 1255 | 298 | 25 | 1195 | 531 |
| **M2** | 3.16 | 1.11 | 1680 | 1520 | 164 | 767 | 771 | 589 | 858 | 102 | 31 | 632 | 518 |
| **M7** | 5.86 | 1.09 | 60 | 55 | 3 | 32 | 30 | 31 | 38 | 6 | 1 | 30 | 17 |
| **M8** | 12.40 | 1.08 | 55 | 51 | 1 | 26 | 29 | 24 | 31 | 5 | 1 | 26 | 12 |
| **M9** | 4.50 | 1.02 | 5 | 5 | 1 | 3 | 3 | 5 | 3 | 5 | 1 | 5 | 5 |
| **M10** | 1.00 | 1.22 | 2 | 2 | 1 | 2 | 1 | 5 | 3 | 5 | 1 | 5 | 1 |
| **GC1** | 5.10 | 0.97 | 547 | 564 | 68 | 306 | 260 | 182 | 317 | 34 | 18 | 217 | 347 |
| **GC2** | 8.75 | 1.03 | 448 | 434 | 71 | 176 | 157 | 116 | 216 | 21 | 140 | 138 | 621 |
| **GC3** | 13.69 | 0.99 | 373 | 376 | 13 | 192 | 112 | 123 | 239 | 27 | 13 | 147 | 178 |
| **GC4** | 5.91 | 1.15 | 23 | 20 | 2 | 16 | 10 | 9 | 21 | 5 | 2 | 12 | 13 |
| **GC5** | 4.44 | 1.13 | 26 | 23 | 3 | 17 | 19 | 22 | 22 | 5 | 2 | 34 | 12 |
| **GC6** | 3.75 | 1.01 | 67 | 66 | 8 | 40 | 52 | 47 | 31 | 13 | 4 | 49 | 31 |
| **GC7** | 5.77 | 1.35 | 36 | 26 | 3 | 14 | 19 | 20 | 34 | 4 | 3 | 25 | 18 |
| **MA1** | 5.32 | 1.25 | 819 | 654 | 145 | 440 | 361 | 319 | 338 | 55 | 46 | 513 | 772 |
| **MA2** | 4.00 | 1.31 | 323 | 247 | 43 | 151 | 139 | 97 | 156 | 15 | 5 | 169 | 173 |
| **MA3** | 9.00 | 1.67 | 10 | 6 | 1 | 3 | 4 | 5 | 6 | 5 | 2 | 9 | 9 |
| **LR1** | 11.77 | 1.04 | 352 | 338 | 51 | 136 | 288 | 207 | 270 | 63 | 406 | 287 | 598 |
| **LR2** | 6.49 | 1.03 | 175 | 170 | 6 | 76 | 67 | 59 | 89 | 42 | 17 | 51 | 41 |

**Supplementary table 1** (continued)

|  | **PAHs concentrations normalized to 1% TOC (ppb)** | | | | | | | | | | |
| --- | --- | --- | --- | --- | --- | --- | --- | --- | --- | --- | --- |
|  | **Fluoranthene** | **Pyrene** | **Anthracene** | **Benz[*a*]anthracene** | **Benzo[*a*]pyrene** | **Benzo[*ghi*]perylene** | **Chrysene** | **Dibenz[*a,h*]anthracene** | **Fluorene** | **Indeno[1,2,3-*cd*]Pyrene** | **Phenanthrene** |
| **BA1** | 38 | 28.6 | 9.7 | 34.3 | 47 | 17.7 | 17.5 | 5.4 | 6.5 | 39 | 26.7 |
| **BA2** | 56.3 | 40.8 | 6.5 | 16.3 | 16.8 | 14.2 | 13 | 2.4 | 3.7 | 16.8 | 33.6 |
| **BA3** | 12.5 | 17.8 | 4.5 | 19.1 | 20.5 | 8.2 | 8.1 | 2.4 | 3 | 16.7 | 13.1 |
| **BA4** | 126 | 145.8 | 38.6 | 132.6 | 112.6 | 44.1 | 45.1 | 11.7 | 21 | 82.2 | 121.2 |
| **BA5** | 9.2 | 7.2 | 1.7 | 6 | 11.4 | 6.9 | 4.6 | 1.4 | 0.9 | 11.6 | 7.9 |
| **PV1** | 123.6 | 103.6 | 22.9 | 152.4 | 187.4 | 63 | 66.9 | 21.4 | 6 | 138.7 | 47.2 |
| **PV2** | 19.8 | 15 | 4 | 17.5 | 19.3 | 9.4 | 6.6 | 3.1 | 1.6 | 20.2 | 9.8 |
| **PV3** | 126.4 | 100.4 | 17 | 111.5 | 151.9 | 57.7 | 40 | 15.3 | 6.6 | 112 | 46.8 |
| **PV4** | 33.9 | 23.4 | 0.3 | 24 | 25.3 | 11.2 | 9.8 | 3.5 | 5.2 | 23.6 | 1.6 |
| **PV5** | 9.1 | 7.4 | 1 | 7.9 | 5.6 | 5.8 | 4.6 | 1.6 | 0.3 | 13.1 | 3.3 |
| **BI1** | 1.5 | 2.6 | 0.5 | 0.4 | 0.7 | 1.6 | 0.3 | 1 | 0.9 | 0.8 | 4.7 |
| **BI2** | 16.1 | 21.8 | 6 | 7.4 | 10.2 | 19 | 5.4 | 4.5 | 13.6 | 17.9 | 28.6 |
| **BI3** | 51.7 | 41.8 | 11.6 | 28.1 | 24.2 | 30.4 | 24.6 | 14.7 | 14.2 | 76.3 | 38.1 |
| **BI4** | 0.7 | 1.1 | 0.1 | 357.9 | 276.9 | 209.6 | 166.3 | 47.4 | 6.9 | 184.1 | 1.3 |
| **L1** | NA | NA | NA | NA | NA | NA | NA | NA | NA | NA | NA |
| **L2** | NA | NA | NA | NA | NA | NA | NA | NA | NA | NA | NA |
| **L3** | 6.9 | 6.4 | 0.8 | 3.5 | 3.3 | 4.8 | 3.8 | 1 | 1 | 5 | 6.5 |
| **M3** | NA | NA | NA | NA | NA | NA | NA | NA | NA | NA | NA |
| **M4** | 258.8 | 211.8 | 18.7 | 146.2 | 166.4 | 120.3 | 157 | 26.9 | 0.2 | 138.2 | 85.9 |
| **M5** | 250 | 223.7 | 29.8 | 130.4 | 166.9 | 131.5 | 161.5 | 33.6 | 12.3 | 135.5 | 118.2 |
| **M6** | 461.2 | 343.1 | 23.5 | 189 | 195.2 | 153.3 | 187.3 | 40.5 | 5 | 204.2 | 93.1 |
| **M11** | 8.3 | 6.9 | 0.8 | 3.9 | 2.5 | 3.7 | 8.3 | 3.5 | 2.5 | 3.5 | 11.1 |
| **M12** | 16.9 | 12.8 | 1.2 | 5.5 | 4.9 | 10.6 | 9.9 | 5.8 | 1.5 | 7.1 | 16.6 |
| **M13** | 8.6 | 7.7 | 0.9 | 4.8 | 5.6 | 5 | 5.4 | 1.3 | 0.6 | 5.7 | 5.1 |
| **T12** | 961.7 | 875.6 | 110 | 413.9 | 442.6 | 325.4 | 468.9 | 35.9 | 52.6 | 461.7 | 612.4 |
| **T15** | 236.4 | 210.1 | 26.3 | 121.2 | 139.4 | 121.2 | 113.1 | 10.1 | 10.1 | 270.7 | 121.2 |
| **T23** | NA | NA | NA | NA | NA | NA | NA | NA | NA | NA | NA |
| **T52** | NA | NA | NA | NA | NA | NA | NA | NA | NA | NA | NA |
| **M1** | NA | NA | NA | NA | NA | NA | NA | NA | NA | NA | NA |
| **M2** | 774.2 | 700.5 | 75.6 | 353.5 | 355.3 | 271.4 | 395.4 | 47 | 14.5 | 291.2 | 238.7 |
| **M7** | NA | NA | NA | NA | NA | NA | NA | NA | NA | NA | NA |
| **M8** | 13.5 | 12.6 | 0.2 | 6.4 | 7.2 | 5.9 | 7.7 | 1.2 | 0.2 | 6.4 | 3.1 |
| **M9** | NA | NA | NA | NA | NA | NA | NA | NA | NA | NA | NA |
| **M10** | 1.8 | 1.4 | 0.8 | 1.7 | 0.8 | 4 | 2.5 | 4 | 0.8 | 4 | 0.8 |
| **GC1** | 701.3 | 723.1 | 87.2 | 392.3 | 333.3 | 233.3 | 406.4 | 43.6 | 23.1 | 278.2 | 444.9 |
| **GC2** | 142.7 | 138.2 | 22.6 | 56.1 | 50 | 36.9 | 68.8 | 6.7 | 44.6 | 43.9 | 197.8 |
| **GC3** | 423.9 | 427.3 | 14.8 | 218.2 | 127.3 | 139.8 | 271.6 | 30.7 | 14.8 | 167 | 202.3 |
| **GC4** | 22.1 | 19.2 | 2.1 | 15.4 | 9.6 | 8.6 | 20.2 | 4.8 | 2.3 | 11.5 | 12.5 |
| **GC5** | 24.5 | 21.7 | 2.5 | 16 | 17.9 | 20.8 | 20.8 | 4.7 | 1.7 | 32.1 | 11.3 |
| **GC6** | 25.5 | 25.3 | 3.1 | 15.1 | 19.8 | 18 | 11.7 | 5.1 | 1.7 | 18.5 | 11.6 |
| **GC7** | 18.6 | 13.7 | 1.6 | 7.1 | 9.8 | 10.6 | 17.5 | 2.1 | 1.3 | 12.9 | 9.3 |
| **MA1** | 920.2 | 734.8 | 162.9 | 494.4 | 405.6 | 358.4 | 379.8 | 62.2 | 51.5 | 576.4 | 867.4 |
| **MA2** | 576.8 | 441.1 | 77.1 | 269.6 | 248.2 | 173.2 | 278.6 | 27.5 | 9.5 | 301.8 | 308.9 |
| **MA3** | 3.7 | 2.2 | 0.4 | 1.1 | 1.5 | 1.9 | 2.2 | 1.9 | 0.7 | 3.4 | 3.4 |
| **LR1** | NA | NA | NA | NA | NA | NA | NA | NA | NA | NA | NA |
| **LR2** | NA | NA | NA | NA | NA | NA | NA | NA | NA | NA | NA |

**Supplementary table 2:** Pyrosequencing dataset description**.**

|  |  | **Bacteria** | **Archaea** | **Eukarya** |
| --- | --- | --- | --- | --- |
| **raw reads** | mean nb. / sample | 15228 | 4822 | 2139 |
|  | total | 639687 | 202519 | 110044 |
| **quality-checked reads** | mean nb. / sample | 9336 | 2730 | 1964 |
|  | total | 392398 | 114654 | 82468 |
|  | mean length (pb) | 273 | 287 | 158 |

**Supplementary table 3:** List of the environmental variables used in analysis, according to Spearman correlations (p<0.05) based on 32 samples.

| Variables used in analysis | Correlated variables \|rho\|>0.75 |
| --- | --- |
| **Temperature** | Temperature |
| **Salinity** | Salinity, Latitude |
| **PSD** | PSD |
| **%TOC** | %TOC |
| **Fluoranthene/Pyrene** | Fluoranthene/Pyrene |
| **Phenanthrene/Anthracene** | Phenanthrene/Anthracene |
| **Dibenz[a,h]Anthracene** | Dibenz[*a,h*]Anthracene, Fluoranthene, Pyrene, Anthracene, Benz[*a*]anthracene, Benzo[*i*]pyrene, Benzo[*g,h,i*]Perylene, Chrysene, Fluorene, Indeno[1,2,3,*c-d*]Pyrene, Phenanthrene |

# Supplementary Figures

**Supplementary figure 1:** Comparisons between Mediterranean and Atlantic sites of the means of the environmental parameters**.** P-values were calculated using the Wilcoxon-Mann-Whitney U test or the Student’s t-Test after testing the homoscedasticity of the data.

**Supplementary figure 2:** Hierarchical classification analysis (HCA) of euclidean distances between the samples based on PAH concentrations at the Mediterranean sea and the Atlantic coasts. Clusters of contaminated (dark grey) and non-contaminated (light grey) samples are indicated for each region.

**Supplementary Figure 3:** Plot of the phenanthrene to anthracene (P/A) ratio against the fluoranthene to pyrene (F/P) ratio. Each dot represents one sediment sample. P/A values above 10 and F/P values below 1 indicate petrogenic input of hydrocarbons. Conversly, P/A values below 10 and F/P values above 1 indicate pyrogenic input of hydrocarbons.

**Supplementary figure 4:** Taxonomic composition of the bacterial (A), archaeal (B) and eukaryotic (C) communities at the class level, showing the taxa with relative abundance up to 5%. The samples were separated between Mediterranean and Atlantic regions, and between non-contaminated and contaminated samples. Bacterial taxonomy was assessed with the Greengenes 2013 database, archaeal taxonomy with the SILVA 119 database and eukaryotic taxonomy was determined using BLAST (see methods).

**Supplementary figure 5:** Taxonomic composition of the Delta-proteobacterial and Gamma-proteobacterial families from the bacterial dataset. Samples were separated between Mediterranean and Atlantic regions, and between contaminated and non-contaminated samples. Bacterial taxonomy was assessed with the Greengenes 2013 database.

**Supplementary figure 6:** Non-metric multidimentional scaling of the Bray-Curtis matrices computed from the bacterial (A) and archaeal (B) metabolic pathways reconstructed using the PICRUSt program. Arrows stand for the significant environmental variables fitted as vectors using the envfit function of the vegan package. ‘Temp’ stands for temperature and ‘Sal’ for salinity.

**Supplementary figure 7:** Non-metric multidimensional scaling plots of the bacterial (A and D), archaeal (B and E) and eukaryotic (C and F) UNIFRAC matrices of the samples from the Mediterranean Sea (A to C) and from the Atlantic coasts (D to F). The colors stand for the areas where the samples come from. Arrows represent the significant environmental variables fitted as vectors using the envfit function of the vegan package.
